# Need for Multilevel Strategies and Enhanced Acceptance of Contraceptive Use in Order to Combat the Spread of HIV/AIDS in a Muslim Society: A Qualitative Study of Young Adults in Urban Karachi, Pakistan

**DOI:** 10.5539/gjhs.v5n5p57

**Published:** 2013-05-27

**Authors:** Syed Farid-ul-Hasnain, Eva Johansson, Saleema Gulzar, Gunilla Krantz

**Affiliations:** 1 Department of Community Health Sciences, Aga Khan University, Karachi, Pakistan; 2 Department of Public Health Sciences, Division of Global Health (IHCAR), Karolinska Institutet, Stockholm, Sweden; 3 Nordic School of Public Health, Gothenburg, Sweden; 4 School of Nursing, Aga Khan University, Karachi, Pakistan; 5 Department of Community Medicine and Public Health, The Sahlgrenska Academy at University of Gothenburg, Gothenburg, Sweden

**Keywords:** young adults, HIV/AIDS, contraceptives, reproductive health

## Abstract

This qualitative study explored knowledge, attitudes, beliefs and perceptions of sexual and reproductive health, focusing specifically on contraceptive use and HIV prevention among young unmarried men and women, 17-21 years, in urban Karachi, Pakistan. The main theme, identified as underlying meaning in the focus group discussions was “Societal norms and perceptions create barriers to knowledge and awareness about sexual and reproductive health matters among young adults”. A knowledge gap was revealed concerning HIV/AIDS and contraceptive use among young males and females, who have to rely on media and peers for information seeking. Study participants perceived that HIV/AIDS is incurable and carries a social stigma. It was further revealed, that there is an opposition towards contraceptive use from religious leaders. Young adults in Pakistan are in need of improved knowledge about HIV/AIDS and contraceptive use. Youth clinics and schools/colleges may play a significant role in this regard. The religious leaders need to be informed about the beneficial effects of contraceptives and they should be part of any family planning/contraceptive use program to ensure better community acceptance. At the structural level there is an urgent need for policies targeting the issue of sexual and reproductive health, particularly HIV/AIDS information and contraceptive use to target the young population. The health care services should be able to respond by offering relevant services.

## 1. Introduction

The sexual and reproductive health concept implies that people are able to have a responsible, satisfying and safe sex life, including the capability to reproduce and the freedom to decide if, when and how often to do so (Reproductive Health; http://www.who.int/topics/reproductive_health/en/). From this follows that women and men should be informed of and have access to safe, effective, affordable and acceptable methods of birth control and access to appropriate health care services to enable women to go safely through pregnancy and childbirth (ICPD, 1994).

Sexual and reproductive health matters are however considered sensitive topics to discuss in several countries and also in Pakistan due to cultural and religious reasons; moreover there is a general lack of information to young people concerning these issues (Ali et al., 2004).

Pakistan is foreseeing its ever-largest younger population because of the high fertility levels over the last few decades augmented by the restricted access and poor knowledge on contraceptive use (Khawaja et al., 2004; National Institute of Population Studies (NIPS) Islamabad Pakistan Macro International Inc. USA, 2008). Pregnancies and births are projected to almost double among adolescent Pakistani girls (10-19 years of age) in the next 20 years (Khan & Pamela, 2003). Most births take place with in marriage, and mean age at marriage has increased from 21.7 in 1990-91 to 23.1 in 2006-07 (National Institute of Population Studies (NIPS) Islamabad Pakistan Macro International Inc. USA June 2008). While overall prevalence of early childbearing is rather low, it varies by education and socioeconomic characteristics (Hanif, 2011; National Institute of Population Studies (NIPS) Islamabad Pakistan Macro International Inc. USA, 2008). About 44 percent of the poorest 20–24 year old women gave birth before reaching 18 years, while only 19 percent of better off women did (World Bank, 2011).

Younger women, even if married, are more restricted than older women in their mobility and access to health care services, including family planning and contraceptive services (Khan, 2000). Women, and specifically poorly educated women, are in general brought up in a society with pronounced gender inequalities with serious restrictions of autonomy and poor possibilities to access any health care services without permission from the husband, resulting in poor health status (T. S. Ali et al., 2011; Rizvi & Nishtar, 2008). Poor education, transport, finances, family's reluctance to bring a woman to hospital, the husband's absence from the house, and inadequate and inappropriate referral services further contribute to an inability to obtain such services (Ladha et al., 2009; Khan, 2000).

The latest demographic survey 2006-7 revealed, that approximately 30% of the currently married women reported use of contraceptives (National Institute of Population Studies (NIPS) Islamabad Pakistan Macro International Inc. USA, 2008). Low contraceptive use is attributable to illiteracy, poverty and lack of awareness about family planning methods (Saleem & Pasha, 2008; National Institute of Population Studies (NIPS) Islamabad Pakistan Macro International Inc. USA, 2008). One study found that both lifetime and current contraception use for the highest as well as the lowest quintile was significantly associated with decision autonomy, further the contraceptive use was strongly associated with women's education (Saleem & Bobak, 2005).

HIV/AIDS is spreading globally, mainly among the younger population (Monasch & Mahy, 2006) and so also in Pakistan (Todd et al., 2007). Heterosexual transmission dominates in Pakistan (Khan & Pamela, 2003) as well as in other Muslim countries like Turkey, Egypt, Malaysia and Iran, but up-to-date the prevalence rates are rather low (Huang & Hussein, 2004; Celikbas et al., 2008; El-Sayyed et al., 2008). According to UNAIDS estimates, the prevalence of HIV/AIDS among men and women aged 15-24 in Pakistan is to date about 0.1% (UNAIDS, 2008). However, figures are unreliable and The World Health Organization (WHO) estimates the total number of children and adults living with HIV has been increased from 79,000 to 150,000 from 2001 to 2007 (World Health Organization, 2008). Pakistan has also entered into a “concentrated epidemic” stage for HIV/AIDS, i.e. the HIV prevalence in high-risk sub-populations is 5% or higher. This indicates that the country is at high risk of a HIV/AIDS epidemic (SACP, 2004). Several socioeconomic factors, such as poverty, migration, and a deep-rooted culture of gender inequality risk aggravating the spread of HIV in Pakistan (Farid-ul-Hasnain et al., 2009; National Institute of Population Studies (NIPS) Islamabad Pakistan Macro International Inc. USA, 2008).

Young people are in their right to make informed decisions for their better sexual and reproductive health, regardless of their cultural morals and societal customs which may condemn these behaviours (Dickens & Cook, 2005). As young adults in Pakistan seldom discuss sexual and reproductive health issues with elders, and hardly any such education is offered at schools, they are at risk of being left with misconceptions and ignorance (Ali et al., 2004).

This study explored knowledge, attitudes, beliefs and perceptions of sexual and reproductive health, focusing specifically on contraceptive use and HIV prevention among young unmarried men and women, 17-21 years, in urban Karachi, Pakistan. Current sources of information on these issues and needs for health education is further discussed.

## 2. Methodology

This qualitative study used focus group discussions (FGDs) for data collection. In FGDs, participants vigorously discuss a specific topic in generating new knowledge about social practices (Krueger, 1994; Morgan, 1997). FGDs have the advantage to utilize group interaction to explore knowledge and perceptions and are well suited for capturing opinions and cultural norms (Dahlgren, 2004).

### 2.1 Study setting and Participants recruitment

The study was carried out in the metropolitan city of Karachi, which is the industrial hub of Pakistan, with a population of 21 million. The participants were purposively chosen to capture as many aspects and views of young adults as possible according to the following criteria: males and females aged 17-21 years, unmarried, living in Karachi and belonging to different social strata. We use the terminology ‘young adults’ and ‘young people’ interchangeably to males and females of this age group, in line with the UN definition of young people covering the age groups 10-24 years (Dehne & Riedner, 2001).

To include rural sites was considered inappropriate due to foreseen community resistance because of cultural reasons and sensitivity of the topic with regards to inquiring young people about such issues.

Six FGDs were performed, one including males and one with females from each social stratum. A community coordinator working in the primary health care centre in *Sultanabad* identified participants belonging to the lowest socio-economic stratum. Participants belonging to the lower-middle socio-economic stratum, (*Qayyumabad*), were identified by the research assistant in this study, whereas the final two FGDs comprising people from the upper middle socio-economic stratum *(Garden East)* were identified through a Non-Governmental Organisation (NGO) ‘Focus Humanitarian Assistance’ working in the area. The participants were approached and asked to participate by the research team members who had good local knowledge in the respective areas. The FGDs from three different strata were carried out at different places either at a nearby health centre, in the house of one of the participants or at the NGO office due to logistic reasons (Table 1).

**Table 1:** Characteristics of the focus-group participants, number of participants and venue for focus group discussions

Position	Gender	No.	Socio economic status (SES)	Venue
**G1**	Males	7	Low SES	Health centre
**G2**	Females	8	Low SES	Health centre
**G3**	Males	8	Lower middle SES	Participants’ house
**G4**	Females	6	Lower middle SES	Participants’ house
**G5**	Males	7	Upper middle SES	NGO office
**G6**	Females	6	Upper middle SES	NGO office

### 2.2 Data Collection

The FGDs were conducted in local language Urdu. Each FGD included 6-8 participants, in all 20 women and 22 men participated.

A topic guide, covering specific areas was developed: a) Perceptions and awareness of sexual and reproductive health matters and contraceptive use, HIV and STDs, phrased as what is important for young people to know today; b) Young adults needs in terms of services, information, and counselling regarding sexual and reproductive health issues.

A pilot-FGD was performed to test and revise the topic guide. The first (SFH) and third author (SG) of this paper moderated the FGDs with men and women respectively. Note taking, tape recording and observations of the participants were undertaken by the research assistant. Each FGD lasted 70-90 minutes.

The tape-recordings were transcribed verbatim into Urdu, supplemented by notes, and later translated into English by the research coordinator who was well versed with both languages. A third person re-examined the translations. Finally the first author (SFH) reviewed all transcribed and translated texts from the recordings and the notes. The data were collected in December 2008.

### 2.3 Analysis

Qualitative content analysis was used, following a well-acknowledged model (Dahlgren, 2004). The manifest content, which is the visible, obvious components was organized into categories. The latent content, which describes the underlying meaning of the text, was discussed between the researchers and phrased as a theme (Graneheim & Lundman, 2004).

The analysis process followed the steps of identifying meaning units in response to the semi-structured issues raised in the FGDs. These were later condensed and codes were identified. The codes were then grouped into categories by three of the researchers (SFH, EJ, GK) independently of each other. Finally one main theme was formulated after discussions on commonalities, variations and disagreements between the three researchers.

### 2.4 Ethical Considerations

Ethical approval for the study was given by The Aga Khan University Ethical Review Committee (AKU-ERC). Informed consent was obtained both from the participants and their parents. The study was well explained with its research objectives to the participants, who were also ensured about the confidentiality and informed about their full right to withdraw at any stage during the study. As the study addressed sensitive topics, the participants were encouraged to only discuss the issues raised in the FGDs in general terms and not to reveal sensitive personal experiences.

## 3. Results

The main theme was identified as “Societal norms and perceptions create barriers to knowledge and awareness about sexual and reproductive health matters among young adults”. The main theme will be described and illustrated below with the help of four categories; Cultural norms and beliefs about reproductive health, Knowledge and awareness about HIV/AIDS, Misconceptions and stigma related to HIV/AIDS and Current and desired sources of information (Table 2).

**Table 2:** Examples of codes, categories and theme from the content analysis of focus group discussions of males and females about HIV/AIDS and contraceptive use

Theme	Societal norms and perceptions create barriers to knowledge and awareness about sexual and reproductive health matters among young adults			
**Category**	Cultural norms and beliefs about contraceptive use	Poor knowledge and awareness about HIV/AIDS	Misconceptions and stigma about HIV/AIDS	Current and desired sources of information
**Code**	Contraceptives are useful Not allowed in religion People dislike Better resource use Difficult to get contraceptives Cultural barriers Lack of availability Religious reasons Seek permission from parents Lack of Female autonomy	Illiteracy Few heard of HIV/AIDS Sexual contact Blood Used syringes Mother to child Condoms Avoidance of sexual contact Incurable disease Westernised behaviour	Social isolation Women blamed more Avoid HIV person Bad character Avoid eating with HIV ^+ve^ person	Television Internet Peers Doctor Parents School/colleges Lady health worker Community health centre

### 3.1 Cultural Norms and Beliefs about Contraceptive use

In response to the question on contraceptives by the moderator; most of the participants discussed child spacing, pregnant women's health and population growth in relation to contraception. Contraceptive use was considered to assist in all these matters. Even on probing there was no mentioning of contraceptives in relation to sexuality, as a means to practice safe sex among younger, unmarried couples.

The young women pointed at serious constraints in relation to contraceptive use due to prevailing norms and culture, such as women's low decision-making power and difficulties in accessing contraceptives for the married couples.

…“In our culture we cannot take the decisions about using contraceptives without asking the elders within the household”. (Female from low SES)

…“Unfortunately in our society, because of cultural reasons, people hesitate to buy contraceptives from stores in the presence of others”. (Male from upper middle SES)

Resistance from religious leaders were also described by the informants as barriers to contraceptive use.

…“Our religious leaders have always disliked contraceptives. The religious leaders perceive that it is Gods’ responsibility to feed and nourish every human being”. (Male from low SES)

### 3.2 Knowledge and Awareness about HIV/AIDS

The informant's knowledge about the spread of HIV/AIDS varied. Most of the participants from the upper middle class were well aware about the modes of HIV spread, described as sexual contacts, used syringes and mother to child transmission, while the majority of young people were considered unaware.

… “As everybody in Pakistan does not go to school/college, they are not educated and have no awareness of HIV/AIDS. We are an educated group who knows about HIV/AIDS”. (Male from upper middle SES)

…“I think most of our young people are unaware, owing to illiteracy. Even those who know have very little knowledge about its modes of transmission”. (Male lower middle SES)

Knowledge about HIV prevention varied among the different social classes especially the use of condoms for STIs prevention. Those who were well educated had more knowledge.

… “We have heard that HIV/AIDS spreads through sexual relations and by using certain measures such as contraceptives (condoms) we can protect ourselves. But there are people who are not aware of HIV/AIDS and contract the disease from sexual contacts”. (Male from upper middle SES)

One female participant pointed at how modern media contributes to increased interaction between young people as a sign of westernised behaviour and what risks this entail.

“Today media, movies and internet have resulted in increased interaction and friendship among girls and boys. They tend to ignore all limitations and boundaries and get inspired from the western society and due to this, sexually transmitted infections are increasing”. (Female from upper middle SES)

### 3.3 Misconceptions and Stigma related to HIV/AIDS

Most of the male and female informants agreed on that the stigma related to HIV/AIDS was a societal disgrace. A male informant stated however his personal opinion:

… “I would treat both a man and a woman, suffering from HIV/AIDS, in the same way but avoid them as much as possible. I can never think of living with an infected person” (Male from low SES)

The young adults expressed that in ‘society’ a HIV/AIDS positive person is considered as having a ‘bad character’ as testing positive is immediately linked to heterosexual spread. Women testing positive were said to be considered inferior and to a higher extent blamed than were infected men and this gender inequality was considered a general notion in society.

…“Generally the society is of the opinion that a girl or a woman (who is HIV infected) is of a bad character, as not everybody knows about the other modes of spread of HIV/AIDS”. (Male from lower middle SES)

… “Our society considers those who are suffering from HIV/AIDS as having a bad personality, people will never sit, eat or have physical contact with them. The society will disgrace a woman more than a man because our culture is like that”. (Female from lower middle SES)

The participants further discussed possible sources of information both current and desired.

### 3.4 Current and Desired Sources of Information

The discussions revealed that young adults in general had poor knowledge about sexual and reproductive health matters but were eager to acquire proper information on these issues, and discussions came to focus on contraceptive access and use, and HIV/AIDS.

The male participants described television, internet and peers as their main sources of information on sexual and reproductive health matters including contraceptives and HIV/AIDS.

… “We come to know about contraceptive use through advertisements on television, i.e. pills. We assume, there may be other methods for contraception, but we know about only one, which is shown on television”. (Male from lower middle SES)

… “I think that the public service messages about HIV/AIDS on television are not clear enough. Only those who know about HIV/AIDS can understand them”. (Male from upper middle SES)

Female participants discussed that mothers, older sisters and married friends were to a certain extent willing to inform about sexual and reproductive health matters, including HIV, however this information was considered to not always be reliable. The females from upper middle SES described doctors and lady health workers as potential sources of information regarding contraceptive use specifically.

As sexual and reproductive health matters are not spoken openly about, some of the male participants described the cultural constraints at hand in acquiring knowledge about HIV/AIDS specifically.

… “If a young boy goes to someone asking about AIDS, people refuse him due to his age. It is difficult to get information from adults due to cultural reasons”. (Male from lower middle SES)

All informants expressed that health care staff and parents should provide information about sexual and reproductive health and HIV/AIDS. Schools and colleges were also considered to have such a responsibility.

…“Doctors in a health clinic is a good source of information about family planning and contraceptives”. (Male from upper middle SES)

…“Well educated teachers at schools and colleges should impart knowledge on reproductive health and HIV/AIDS. It should also be made a compulsory topic at school/colleges”. (Female from upper middle SES)

Moreover, participants from lower SES suggested workshops and meetings in the communities as a proper source of HIV/AIDS information as not all young people attended school or are able to access to health care services.

## 4. Discussion

The findings of this study point that the growing younger generation being seriously uninformed on sexual and reproductive health matters and denied knowledge and skills on these matters. It seems young people's right to acquire knowledge and skills to protect themselves and their partners from HIV, unwanted pregnancies, unsafe abortions and also sexual abuse or violence are ignored.

The participants expressed that poor and lower educated young people had less awareness on reproductive health, contraceptive use and HIV prevention than the more educated. Gender discrimination in access and use of contraceptives and serious stigma towards HIV infected people, and especially women, were further revealed. This young population was worried about the present state of information accessible to them, such as from peers, different internet sources and TV commercials, but eager to access proper information on these issues in the first case from health care workers, parents and teachers.

### 4.1 Our Findings in Relation to Other Studies

The reluctance in the Pakistani society to discuss sexuality, contraceptive use and how to protect oneself from STIs including HIV are well-known (Kapamadzija et al., 2000; Ali et al., 2004). The findings that contraceptives were mainly discussed in terms of slowing population growth to make the country prosperous and to prevent STIs are found also in other studies (Anderson et al., 2008; Kandwal & Bahl, 2011). Religious leaders further opposed contraceptive use, which is also noted in other study (National Institute of Population Studies (NIPS) Islamabad Pakistan Macro International Inc. USA, 2008). Islam is however not in general against contraceptive use, as evident from the involvement of religious leaders in the Islamic Republic of Iran, leading to a successful family planning program (Hoodfar & Assadpour, 2000).

The need to open up between generations and in society at large for discussions on sexual and reproductive health issues was well illustrated by the communication problems with elders that the younger generation experienced. Feelings of uneasiness and embarrassment arose when such matters were brought up and the young adults also feared harassment and violence from the elder generation.

Women's need to seek husband's permission to use contraceptives are examples of gender inequality and lack of female autonomy, described also in other study (Mahmood & Ringheim, 1996). Interestingly however, a study that discuss the concept of female autonomy in depth conclude that uptake of reproductive health services as an indicator of autonomy is an erroneous assumption due to failure to explore the interplay of other disadvantages, such as caste, class and socio-economic position (Mumtaz & Salway, 2009). This study further argues that women who report discussing family planning with their mother’s-in-law are more likely to use a modern contraceptive method. This study obviously views such bonds between women as a social resource but disregard it as female autonomy. This distinction builds on female autonomy as related only to individual power, while we believe social network should be viewed as part of female autonomy.

The informants described HIV/AIDS in relation to social stigma, which is a global phenomenon as evident from various studies (Anderson et al., 2008; Winskell et al., 2011; Pharris et al., 2011). The female participants further claimed that the stigma was more pronounced towards women infected by HIV, and interpreted this as part of the current gender discrimination and women's lower status in society. Inadequate knowledge about HIV/AIDS and prevailing myths and misconceptions about the spread, such as to avoid eating with a HIV infected person and to use separate towels are detrimental as it will add to the stigma and make preventive work more difficult (Fox et al., 2010; Kandwal & Bahl, 2011).

Current and desired sources of information were eagerly discussed. Young males mostly acquired information about HIV/AIDS from friends, media and internet sources, and not from parents. This finding is consistent with another study on male adolescents in a rural district of Pakistan, which revealed poor parental communication and how adolescents relied on friends and media for seeking such information (Ali et al., 2004). Young females however also received some limited information from their mothers and elderly sisters, which is reflective of cultural values in Pakistan, where females are more close to their mothers while fathers are left out of such discussions.

Information from public service, internet sources or peers were considered to be selective and lack clarity, further it might be incorrect or inadequate as also stated in another study (Kapamadzija et al., 2000). Policy makers should consider whether these are reliable sources of information for young people to lean on.

The participants desired schools/colleges to incorporate information on sexual and reproductive health matters into the curriculum. This is a relevant suggestion as it demands no active information seeking through clinics or other sources. A study performed in Karnataka, India, revealed that an educational intervention program regarding reproductive health issues can bring about a desirable change in knowledge among adolescent girls (Rao et al., 2008). Furthermore, participants unanimously desired doctors, nurses and parents to impart knowledge and information about HIV/AIDS. Unfortunately, few NGOs in Pakistan are engaged in youth clinics which would otherwise be an appropriate source. However, privacy and confidentiality must be ensured or else young people might not be comfortable to visit such clinics as has been shown in studies from Sri Lanka and USA (Lichtenstein, 2003; Tylee et al., 2007; Agampodi et al., 2008).

### 4.2 Methodological Aspects

The idea of bringing young people together in focus group discussions was to explore their knowledge, attitudes and perceptions about sexual and reproductive health matters but came to embrace mainly contraceptive use and HIV as the participants steered the discussion in response to questions raised by the moderator. Contraceptives were discussed in relation to child spacing and to reduce population growth while sexuality was not touched upon. This might be due to the participants’ young age, not yet married and without children, afraid of becoming pregnant or contracting an STI and the taboo tied to sexual matters.

The qualitative approach using FGDs ensured better understanding and perceptions of young men and women about the sensitive topics of HIV/AIDS and contraceptive use (Patton, 1999); because this was a feasible strategy to gather their views in groups, as participants may feel more confident to discuss such sensitive topics surrounded by peers rather than by being interviewed individually (Dahlgren, 2004). The moderator phrased the issues in terms of how ‘young people think about’ to avoid any personal sharing of information. This study did not enroll participants from the higher SES as it was almost impossible to approach them due to resistance from the community.

#### 4.2.1 Trustworthiness

Triangulation of researchers in analysing data and sharing the preliminary finding with the colleagues to streamline the research process increases the credibility of study (Dahlgren, 2004), as the insiders’ perspective was further broadened by the outsiders’ views. The findings were also shared with the participants later in a meeting and they approved of the findings, which also ensured credibility of the study (Dahlgren, 2004). The quotations given in the study are proposed to facilitate the reader's evaluation of the credibility of results (Graneheim & Lundman, 2004). Moreover, these findings are supported by results obtained in one of our previous studies, a quantitative study on young adults (17-21 years) in Karachi, Pakistan (Farid-ul-Hasnain et al., 2009), which also ensured credibility (Dahlgren, 2004).

As with qualitative studies in general, it is difficult to generalize the findings (Khan et al., 1991). However, the selection of participants with both males and females of varying age groups from different parts of Karachi and from different social strata, make it probable that the findings are applicable to similar population groups. Moreover, carefully conducted and analysed FGDs may offer the possibility to generalize the results to other population groups with similar characteristics (Krueger, 1994; Kitzinger, 1995). Dependability and conformability of the study was ensured by following a ‘decision trail’ by sharing the analysis plan and the citations with another researcher not involved in the study (Dahlgren, 2004), and asked to combine the selected citations and also to the sub-categories, categories and main theme. As there was congruence between researchers within and outside of the study, the procedure strengthens trustworthiness of the study.

## 5. Conclusions

Pakistan is within the coming 20 years foreseeing its largest population of young people in history. The existing situation of denying young adults appropriate and timely information on sexual and reproductive health issues will threaten the health of this young population. Interaction between the sexes is growing due to forces related to globalization.

Young adults need access to quality clinical services able to offer effective treatments and vaccines, coupled with sex education that gives medically accurate information tailored for this age group. Along with these two most important components, young people also need supportive adults for guidance on sexual and reproductive health matters and educational and economic opportunities.

There is an urgent need in Pakistan to develop public health strategies and curriculum based education programs not only targeting individual behaviors, but also address social contexts and structural factors that act against safe sex. Evidence exists on the effectiveness of such programs and small-scale initiatives have been taken by non-governmental organizations in Pakistan. Religious leaders need to be made aware and involved in policy formulation as they can play a major role in propagating family planning and STI prevention.
